# Characterization of the aromatic profile of Ruché wine from Piedmont (Italy) with gas chromatography–mass spectrometry and unsupervised machine learning techniques

**DOI:** 10.1002/jsfa.14083

**Published:** 2024-12-20

**Authors:** Ciro Orecchio, Roberto Rabezzana, Marco Vincenti, Elisabetta Bonometti, Enzo Laurenti, Lorenza Operti, Monica Rigoletto

**Affiliations:** ^1^ Department of Chemistry University of Turin Turin Italy

**Keywords:** Ruché, volatile organic compounds, HS‐SPME, GC‐MS, Chemometrics

## Abstract

**BACKGROUND:**

The volatile composition of Ruché, a red wine produced from a native grape in Piedmont (Italy), was investigated using headspace‐solid phase microextraction (SPME) coupled to gas chromatography–mass spectrometry (GC/MS). The main volatile compounds of the wine were identified and quantified. Chemometric techniques were applied to identify features and possible clusters among the different samples.

**RESULTS:**

Forty volatile compounds were unambiguously identified in the 36 wine samples from different producers. The aroma profile was mainly composed of different alcohols and esters but also featured appreciable concentrations of terpenes, aldehydes, and octanoic acid. 2‐Methyl benzaldehyde was identified for the first time. A high concentration of isoamyl alcohol significantly contributed to the aroma complexity. Differences between producers are highlighted.

**CONCLUSION:**

The present work is the first report about the volatile profile of Ruché wines investigated by chemometric methods with quantitative results. Multivariate exploratory approaches revealed minor but distinct differences among the studied wines, likely attributable to variations in winemaking procedures. This study could be developed in future by investigating possible differences between wines according to different production vintages. © 2024 The Author(s). *Journal of the Science of Food and Agriculture* published by John Wiley & Sons Ltd on behalf of Society of Chemical Industry.

## INTRODUCTION

Ruché is a red wine produced from a native grape variety in a very restricted area of Piedmont, a region in north‐west Italy. According to production regulations, the Ruché vine can be cultivated in the territory of seven villages only (Castagnole Monferrato, Montemagno, Portacomaro, Refrancore, Scurzolengo, Viarigi, Grana). Among them, the most important is by far Castagnole Monferrato, where most of the wine makers (≈40) are located. This is why the full official name of the denomination of controlled and guaranteed origin (DOCG), officially recognized in 2010 and equivalent to protected designation of origin (PDO) under European regulations, is ‘Ruché of Castagnole Monferrato’. Despite the very limited territory in which the wine is produced, the popularity of Ruché wine has resulted in a rapid increase in the vineyard area, which has expanded from the original 10^2^ km^2^ (≈25 acres) in 1988, to about 10^3^ km^2^ (≈250 acres) in 2010, further doubling to 2.04 × 10^3^ km^2^ in 2022, when 1.1 million bottles were produced.

Piedmont is one of the most important wine‐producing regions in Italy, and Ruché is one of the 17 wines from Piedmont to hold the DOCG denomination.^1^ The Ruché vine has been known since ancient times. Indeed, various folkloristic stories arose around its denomination: some studies attribute its name to the vineyards’ proximity to a Benedictine convent dedicated to Saint ‘Rocco’, subsequently destroyed, while others relate its name to the vine's predilection for the steepest and sunniest ‘rocks’. Despite its ancient origin, it was only in the early 1980's when the wine was acknowledged with the Denomination of Controlled Origin (DOC). Finally, due to a production regulation of 2010, Ruché effectively became the smallest DOCG in the Monferrato area in terms of area of production.

Ruché wine features a specific aromatic flavor, where floral nuances are predominant. Bonino *et al*.^2^ investigated their aromatic composition, focusing on the primary aromatic substances – those directly deriving from the grape. Their analyses were conducted by headspace solid phase micro extraction (HS‐SPME) coupled with gas‐chromatography and mass spectrometry (GC–MS). According to their results, the primary aroma profile of Ruché shows some similarities with Malvasia, a wine produced from an aromatic vine. Subsequently, Genovese *et al*.^3^ employed a solvent extraction procedure to perform gas chromatography‐olfactometry and GC–MS analysis on a Ruché wine extract, with the aim of identifying the main analytes responsible for the perceived fragrances. Their results agreed with those of Bonino *et al*.^2^ in highlighting the role played by varietal aromas.

The purpose of the present paper was to study the whole aromatic composition of Ruché wine and to compare the composition of Ruché wines from different producers to verify the variability of Ruché components. It is well known that the aromatic profile of a wine could be adopted as a scientific basis for wine classification and for characterization of typical wine varietals, their provenance, soil composition, and aging conditions, as volatile organic compounds (VOCs) are closely related to the *bouquet* – that is, the characteristic and peculiar fragrance of wine.^4^, ^5^ Moreover, the study of aromatic compounds may help to improve the wine quality during the various steps of vine cultivation and wine processing. The analytical measurements were performed using HS‐SPME coupled with GC–MS. Solid‐phase micro extraction (SPME) is a fast, robust and unexpensive technique,^6^ which has been used consistently for the characterization of several wine varieties and the analysis of their flavor components,^7^, ^8^, ^9^ even if the SPME fiber can capture only a small amount of volatile substances, resulting in a limited sensitivity, especially toward the most polar flavor components such as some terpene derivatives. The experimental data gathered in this work were processed by traditional and advanced exploratory chemometric techniques to hypothesize possible clusters and peculiar features occurring among the investigated samples.

## MATERIALS AND METHODS

### Wine samples

Thirty‐eight wine samples from different producers were purchased directly from the wine makers or in different shops and markets. Thirty‐one wine samples were from the 2021 vintage, but this vintage was unavailable from some producers. Consequently, wines from other vintages were included: 2019 (two wines), 2020 (three), and 2022 (two). As noted, most producers are located in the Castagnole Monferrato territory, accounting for 22 of the 36 wines (labeled CM, n. 22). The remaining 14 wines were sourced from the other four villages permitted to cultivate the Ruché vine under its production regulations: Grana (GR, *n* = 4), Montemagno (MO, *n* = 4), Portacomaro (PO, *n* = 3), and Viarigi (VI, *n* = 3). All these villages lie within the Asti region (44° N, 8° E), which has an average annual rainfall of 900 mm, average humidity of 74%, and average solar radiation of 5.5 MJ·m^−2^. Forests cover approximately 26% of the territory, with an average altitude of 240 m above sea level.

Table 1 reports the list of the wines studied with the labels employed throughout the paper to identify them, the year, and the site of production. All the wine bottles displayed the DOCG certification mark, as a warranty of quality and geographical origin. Winery identities are not reported in order to protect proprietary interests. The average alcohol content of wines was 15% v/v. For all wines, fermentation and ageing were conducted in stainless steel. All samples were stored at 4 °C until analysis.

**Table 1:** Wine samples

Year	CM	GR	MO	PO	VI
2019	ru25				
2020			ru20		ru07, ru11
2021	ru01, ru02, ru03, ru06, ru09, ru12, ru15, ru16, ru18, ru19, ru22, ru23, ru26, ru27, ru28, ru29, ru35, ru37, ru38, ru40, ru42	ru17, ru21, ru24, ru41	ru10, ru39	ru08, ru13, ru14	ru05
2022			ru04		

CM, Castagnole Monferrato; GR, Grana; MO, Montemagno; PO, Portacomaro; VI, Viarigi.

### Reagents and standards

All analytical standards, 3,4‐dimethylbenzaldehyde (used as the internal standard), and *n*‐alkane mixture (C_8_–C_20_) (used for determining the retention indexes), were of analytical purity and were purchased from Sigma‐Aldrich (St Louis, MO, USA) and used as received. For headspace sampling, 5 mL wine aliquots were placed in a 20 mL glass vial (headspace volume 15 mL) with 1.0 g of sodium chloride and 5 μL of a 3,4‐dimethylbenzaldehyde ethanolic solution (0.2 g kg^−1^, 2 × 10^−4^ g kg^−1^ in the sample). The vial was sealed with an aluminium‐coated silicone rubber septum and conditioned at 40 °C for 45 min before sampling. Headspace extraction was performed with a divinylbenzene/carboxen/polydimethylsiloxane (CAR/PDMS/DVB) 50/30 μm fiber from Supelco (Bellefonte, PA, USA) for 60 min at 40 °C under continuous stirring. Then, the fiber was transferred into the gas chromatography (GC) inlet. Extraction time and temperature were selected after extensive testing until optimal reproducibility was reached. Each experiment and analysis was conducted in triplicate.

### Gas chromatography–mass spectrometry

A Focus GC Thermo Scientific (Waltham, MA, USA) gas chromatograph with quadrupole mass spectrometer, operated in electron ionization (EI – 70 eV) mode, was used for measurements. The GC system was equipped with a Supelcowax fused silica capillary column (10, 30 m × 0.25 mm ID; 0.25 μm film thickness) from Supelco. Gas chromatography conditions were as follows: inlet temperature, 200 °C; splitless time, 3 min; oven program, initial temperature 40 °C (held for 3 min), increased to 160 °C at 2.0 °C·min⁻¹ (held for 1 min), then to 200 °C at 10 °C·min⁻¹ (held for 2 min); GC–MS transfer line temperature, 250 °C; carrier gas, helium (purity grade 5.5) at 1.2 mL·min^−1^. The MS ion source temperature was kept at 250 °C. Mass spectra and reconstructed total ion chromatograms (TIC) were obtained by automatic scanning in the *m/z* 35–600 mass range. Gas chromatography–mass spectrometry data were processed with Excalibur 1.4 software. Blank fiber runs were performed after every ten analyses to check for memory effects. Volatile organic compounds were identified by comparing mass spectra with those in the National Institute of Standards and Technology (NIST) mass spectral library (version 98, match probability > 90%) and by comparing experimental retention indexes with theoretical values. Experimental indexes were determined as described by Golovnya *et al*.,^10^ by injecting a *n*‐alkane mixture (C_8_–C_20_) into the chromatograph. Theoretical indexes were derived from the NIST 98 library. In doubtful cases, injection of pure standards was performed. Quantification was carried out by the internal standard method. For this purpose, eight different standard solutions were prepared by dissolving exact volumes of the analytical standard compounds, listed in Table 2, in 12.0% v/v ethanol solutions at 0.1 g kg^−1^ concentration. Next, a set of six standard mixtures containing known concentrations of the chemical standards were prepared by dilution. Then 6 × 10^−3^ g cm^−3^ of tartaric acid and the internal standard (IS, at the same concentration as the samples) were added to every synthetic standard solution. The standard mixtures were analyzed and a six‐point calibration graph of relative component area (A_analyte_/A_IS_) versus analyte concentration (C_analyte_) was drawn to confirm a linear detector response, and from which the amount of the analyte could be determined. Each chemical standard was used to quantify several compounds with similar chemical structure (Table 2).

**Table 2:** List of analytical standards used and molecules quantified by each standard

Analytical standards	Components considered in quantitative determinations
1‐butanol, 3‐methyl	Aliphatic alcohols
1‐butanol, 3‐methyl acetate	Acetates
Benzaldehyde	Aldehydes
Diethyl succinate	Diethyl succinate
Ethyl octanoate	Esters of fatty acids
Octanoic acid	Octanoic acid
Phenyl ethyl alcohol	Phenyl ethyl alcohol
Terpineol	Terpenes

### Chemometrics

The data analysis was performed with Python programming language.^11^ The adopted version (3.11.9) provides essential packages and libraries, including Numpy^12^ for scientific computing, Pandas^13^ for data structuring and manipulation, Matplotlib^14^ and Plotly for graphical outcomes, and Scikit‐learn^15^ for machine‐learning implementations. Various unsupervised techniques were applied to the data, including principal component analysis (PCA), hierarchical cluster analysis (HCA), and *t*‐distributed stochastic neighbor embedding (t‐SNE). All the data were auto‐scaled before performing any type of unsupervised analysis.

## RESULTS AND DISCUSSION

### Volatile compounds

Forty compounds were identified unambiguously in the volatile fraction of Ruché wines. However, 13 of these compounds showed very low concentrations and were present in a few samples only; they were therefore not included in the following discussion. Among the analytes discarded, three varietal compounds were included, namely nerol oxide, terpinene‐4‐ol, and hotrienol. They all belong to the class of terpene derivatives, which generally display very low olfactory perception thresholds and feature fruity and floral notes. It is therefore conceivable that they contributed to the Ruché *bouquet* to an extent comparable to those of analytes detected in higher abundance but their detected concentration was possibly underestimated, because the extraction of such polar compounds was likely to have been incomplete on the apolar fiber coating we adopted.

Table 3 reports the 27 analytes considered in the subsequent elaborations with their mean concentrations (g kg^−1^) and relative standard deviations. The compounds are listed according to their chemical class. Table 3 also reports the odor thresholds (OTH) and odor activity values (OAV).

**Table 3:** Volatile composition of Ruché wines: concentration (g kg^−1^, mean ± standard deviation), calculated and theoretical retention indexes, odor threshold (OTH), and odor activity (OAV) values

Compound	Mean concentration (g kg^−1^)	Standard deviation	Calculated retention index	Theoretical retention index	OTH (g kg^−1^)	OAV^a^
Esters						
1‐butanol, 3‐methyl‐, acetate	2.31 × 10^−3^	6.4	1123	1122	3 × 10^−5^ ^b^	77
Acetic acid, 2‐phenylethyl ester	4.3 × 10^−4^	0.3	1812	1813	2.5 × 10^−4^ ^b^	1.7
Benzeneacetic acid, ethyl ester	1.1 × 10^−4^	0.1	1783	1783	7.3 × 10^−5^ ^c^	1.5
Benzoic acid, methyl ester	1.7 × 10^−4^	0.3	1615	1612	3 × 10^−5^ ^d^	5.7
Butanedioic acid, diethyl ester	1.761 × 10^−2^	10.8	1681	1680	2.00 × 10^−1^ ^b^	0.088
Decanoic acid, ethyl ester	4.48 × 10^−3^	5.0	1639	1638	2 × 10^−4^ ^b^	22.4
Dodecanoic acid, ethyl ester	9 × 10^−5^	0.2	1853	1841	1.5 × 10^−3^ ^b^	0.06
Ethyl acetate	1.50 × 10^−3^	1.5			7.5 × 10^−3^ ^b^	0.2
Ethyl lactate	1.71 × 10^−3^	1.2	1348	1347	1.4 × 10^−2^ ^b^	0.12
Hexanoic acid, ethyl ester	8.13 × 10^−3^	9.5	1236	1233	1.4 × 10^−5^ ^b^	581
Nonanoic acid, ethyl ester	1.1 × 10^−4^	0.2	1539	1531	1.3 × 10^−3^ ^e^	0.085
Octanoic acid, ethyl ester	2.426 × 10^−2^	25.5	1436	1435	5 × 10^−6^ ^b^	4852
*Subtotal*	6.091 × 10^−2^					
*%*	1.028 × 10^−2^					
Alcohols						
1‐butanol, 3‐methyl‐	4.5505 × 10^−1^	246.2	1216	1209	3.0 × 10^−2^ ^b^	15.2
1‐heptanol	1.27 × 10^−3^	1.1	1458	1453	3 × 10^−4^ ^b^	4.2
1‐hexanol	7.91 × 10^−3^	3.7	1355	1355	8 × 10^−3^ ^b^	0.99
1‐hexanol, 2‐ethyl‐	5.1 × 10^−4^	1.1	1488	1491	N/A^f^	
1‐nonanol	7.4 × 10^−4^	0.8	1662	1660	N/A^f^	14.8
1‐octanol	1.05 × 10^−3^	0.7	1560	1557	9 × 10^−4^ ^g^	1.2
1‐propanol, 2‐methyl‐	3.74 × 10^−3^	12.2	1115	1092	4.0 × 10^−2^ ^b^	0.094
Phenylethyl alcohol	5.778 × 10^−1^	39.3	1907	1906	1.4 × 10^−2^ ^b^	4.12
*Subtotal*	5.2805 × 10^−1^					
*%*	8.910 × 10^−1^					
Terpenes						
Citronellol	3.0 × 10^−4^	0.2	1769	1765	1 × 10^−4^ ^h^	3
Linalool	5.3 × 10^−4^	0.3	1552	1547	2.5 × 10^−5^ ^b^	21.2
α‐terpineol	4.3 × 10^−4^	0.2	1694	1697	3.3 × 10^−4^ ^b^	1.3
*Subtotal*	1.26 × 10^−3^					
*%*	2.1 × 10^−4^					
Aldehydes						
Benzaldehyde	3.3 × 10^−4^	0.5	1492	1520	3.5 × 10^−4^ ^i^	0.94
Benzaldehyde, 2‐methyl‐	1.09 × 10^−3^	1.6	1638	1632	N/A^f^	
Furfural	2.7 × 10^−4^	0.4	1466	1461	1.41 × 10^−2^ ^g^	0.019
*Subtotal*	1.68 × 10^−3^					
*%*	2.8 × 10^−4^					
Carboxylic acids						
Octanoic acid	7.4 × 10^−4^	0.6			5 × 10^−4^ ^g^	1.5
*%*	1.2 × 10^−4^					

^a^ Calculated as the ratio concentration/odor threshold value.

^b^ Retrieved from Avellone *et al*.^7^

^c^ Retrieved from Tat *et al*.^36^

^d^ Retrieved from Aznar *et al*.^32^

^e^ Retrieved from Arcari *et al*.^37^

^f^ N/A = not available.

^g^ Retrieved from Verzera *et al*.^38^

^h^ Retrieved from Guth^39^

^i^ Retrieved from Tufariello *et al*.^40^

In general, the triphasic fiber coating Carboxen/divinylbenzene/polydimethylsiloxane (CAR/DVB/PDMS) is largely adopted in food matrix sampling, including wines,^2^, ^7^, ^8^, ^9^ because of its versatility, as it may extract a broad range of analytes with different chemical structures. The extraction efficiency of CAR/DVB/PDMS fibers for the most polar wine volatiles, such as alcoholic terpene derivatives, is modest, limiting their detection to only a few samples and preventing the data from reaching statistical significance. The statistical analysis in our study was therefore focused mainly on the fermentative and aging varietal compounds, mostly consisting of slightly polar esters and higher alcohols, which were detected in appreciable abundance.

Higher alcohols represent the most abundant components, as they amount to about 80% of the total concentration of volatile organic compounds. Among them, 3‐methyl‐1‐butanol (isoamyl alcohol) represents by far the most abundant analyte with an average concentration above 0.4 g kg^−1^. Isoamyl alcohol is the result of leucine and valine metabolism but can also be formed from pyruvate.^16^

The second most abundant alcohol is 2‐phenylethanol, which is produced through the Ehrilch pathway starting from 2‐phenylalanine.^17^ It contributes to the floral nuance of Ruché wine but is also present in most wine varieties and could not be considered peculiar to Ruché. The detected concentration of 2‐phenylethanol greatly exceeded the OAV value of 1, indicating its important contribution to the Ruché aroma. Generally, moderate concentrations of some volatiles with high fragrance intensity, such as isoamyl alcohol and 2‐phenylethanol, give the wine positive sensory features, conferring flower, honey, and fruit aroma notes. However, this typically occurs when their concentrations do not exceed 0.3–0.35 g kg^−1^, whereas, according to some authors,^18^, ^19^ higher alcohol concentrations become detrimental for the wine aroma when they exceed 0.4 g kg^−1^, as they add pungent and unpleasant hints, making the ‘spirit’ nuance too strong and covering other aromas.

The third most abundant alcohol is 1‐hexanol, which is reported to produce a herbaceous scent. In Ruché, its OAV does not exceed 1, making it perceptible but not detrimental to the overall wine aroma. The C_7_–C_9_ linear alcohols are also present in low concentrations. Like hexanol, they are formed by decarboxylation of fatty acids^20^ and they display fruity aromas. Their concentration in wines normally decreases with aging due to esterification reactions.

The second most abundant class of Ruché components is represented by esters. They are scarcely present in grapes but are generated during fermentation by enzymatic activity and with aging. In particular, esterification reactions take place during storage with formation of a variety of esters that contribute to the wine aroma. The most abundant ester in Ruché wine is diethyl succinate; it features a fruity odor (watermelon) but combined with a high perception threshold, resulting in an OAV lower than 1. It is considered an aging ester^17^ – that is, it is formed during the second fermentation of wine. Ethyl lactate is also formed during malo‐lactic fermentation; despite being quite abundant, it also displays a high odor threshold that makes its OAV lower than 1. Ethyl octanoate is also quite abundant in Ruché wine and imparts fruity and ethereal odors.^20^ Acetic esters are formed by the reaction of coenzyme acyl‐S‐CoA with higher alcohols.^16^ The most abundant ones in Ruché are isoamyl acetate (banana scent) and phenylethyl acetate (floral scent), both arising from the alcohols with the highest concentration. The esters mentioned in this paragraph are quite common in red wines and, therefore, cannot be considered as peculiarly responsible for the uniqueness of Ruché *bouquet*. Indeed, this is largely ascribed to varietal compounds, and only the esters featuring OAVs higher than 1 play a significant role in the overall wine aroma.

Aldehydes only account for 0.28% of total aromatic composition. These compounds are not very abundant in grapes but they are formed in wine through different pathways.^16^ Benzaldehyde is a product of fermentation, but it could also originate from *Botrytis cinerea* spoilage.^21^ At low concentrations, it imparts a pleasant oak flavor, but when its concentration is too high, an unpleasant ‘jam’ aroma is perceived, thus representing a negative factor. The presence of furfural in wine may also be ascribed to *Botrytis cinerea* spoilage.^16^ However, its average concentration is quite low and its OAV far lower than 1. 2‐Methylbenzaldehyde is another component of Ruché, which, as far as the authors are aware, has never been detected before in wine. This compound was reported in lamb meat extracts^22^ and it is also a component of Swiss cheese flavor.^23^ Its formation could begin with the complexation of proline and ethanal. As proline is one of the most abundant amino acids in must^16^ and ethanal results from ethanol oxidation, this hypothesis presents a plausible pathway for 2‐methylbenzaldehyde formation in wine. Nevertheless, it cannot be considered as a characteristic marker of Ruché wine as it is not present in all the samples examined.

Terpenes are varietal aromas present in the skin of aromatic vines and are transferred to wine during various production steps. Bonino *et al*.^2^ stated that Ruché shares several features with aromatic wines, despite being produced from a nonaromatic vine, because numerous terpenes and other varietal aromas were detected in its volatile composition. However, the wines under examination were produced by micro‐vinification by the authors, and were not commercial products. As mentioned above, the intrinsic limitations of the adopted CAR/PDMS/DVB fibers in extracting polar substances allowed us to detect only three terpene derivatives, present in most samples at appreciable concentrations, namely citronellol, linalool, and terpineol. As outlined at the beginning of the discussion section, three further terpene derivatives were detected, but only in a few samples. Overall, the presence of varietal compounds such as terpenes is crucial in defining the distinctive aromatic profile of a wine, even if they are present in low concentrations, because they are mostly characterized by low detection thresholds. Among the three most abundant monoterpenes, citronellol and terpineol displayed OAV values above 1. These are among the most odoriferous compounds and impart lemon and rose scent, respectively. However, the role played by the aromatic compounds is synergistic and it is therefore conceivable that the less represented varietal aromas contribute effectively to the complexity of the Ruché *bouquet*.

Only one fatty acid was detected in relatively high concentration, namely caprylic (octanoic) acid. Fatty acids in wine are produced by yeast. Fatty acids hinder fermentation when their concentration is too high (above 1 × 10^−3^ g kg^−1^)^16^ and they may be added deliberately to the must with the purpose of producing sweet wines.^24^ This addition also has the effect of reducing the amount of SO_2_ needed. However, the average concentration found in Ruché wines was below 1 × 10^−3^ g kg^−1^.

Overall, the results generally agree with those of Genovese *et al*.^3^ regarding the identification of the most abundant volatile substances. However, the solvent extraction procedure employed by Genovese allowed the determination of a higher number of analytes, especially among the class of varietal compounds, which play a major role in the definition of the wine aroma profile, such as geraniol, geranic acid, and *β*‐damascenone. Nevertheless, SPME is nowadays largely employed in food aroma determination because of its advantages, including the avoidance of halogenated solvents and ease of use. Detection of polar analytes such as terpenols could be improved by employing different fiber coatings, which exhibit different selectivity profiles.

### Unsupervised methods

In the studies involving the comparison of several samples, a large amount of data is generated by the modern analytical techniques and the adoption of multivariate statistical approaches is mandatory to exploit the useful information potential present in the chemical data and gain a deeper understanding of complex chemical systems.^25^, ^26^ In the present study, all wine samples were produced in a restricted area from the same native grape variety, suggesting a relative uniformity in their composition and no predetermined class separation. The data were therefore studied by several multivariate data exploration techniques, including the well‐known PCA^27^ and HCA,^28^ together with a more recent unsupervised method, namely t‐SNE,^29^ in order to investigate in detail the differences and similarities occurring among the assorted wines productions and the possible correlations among the substances that define their sensory properties.

The less‐known t‐SNE technique deserves some description. It is a nonlinear and manifold‐based machine‐learning approach, particularly suitable for embedding *p*‐dimensional data in a two‐ or three‐dimensional space while preserving the significant structure of the original data.^30^ This SNE‐based algorithm converts the high‐dimensional Euclidean distance between samples into conditional probabilities, which can be interpreted as similarity values.^29^

### Multivariate data analysis

**Figure 1:**
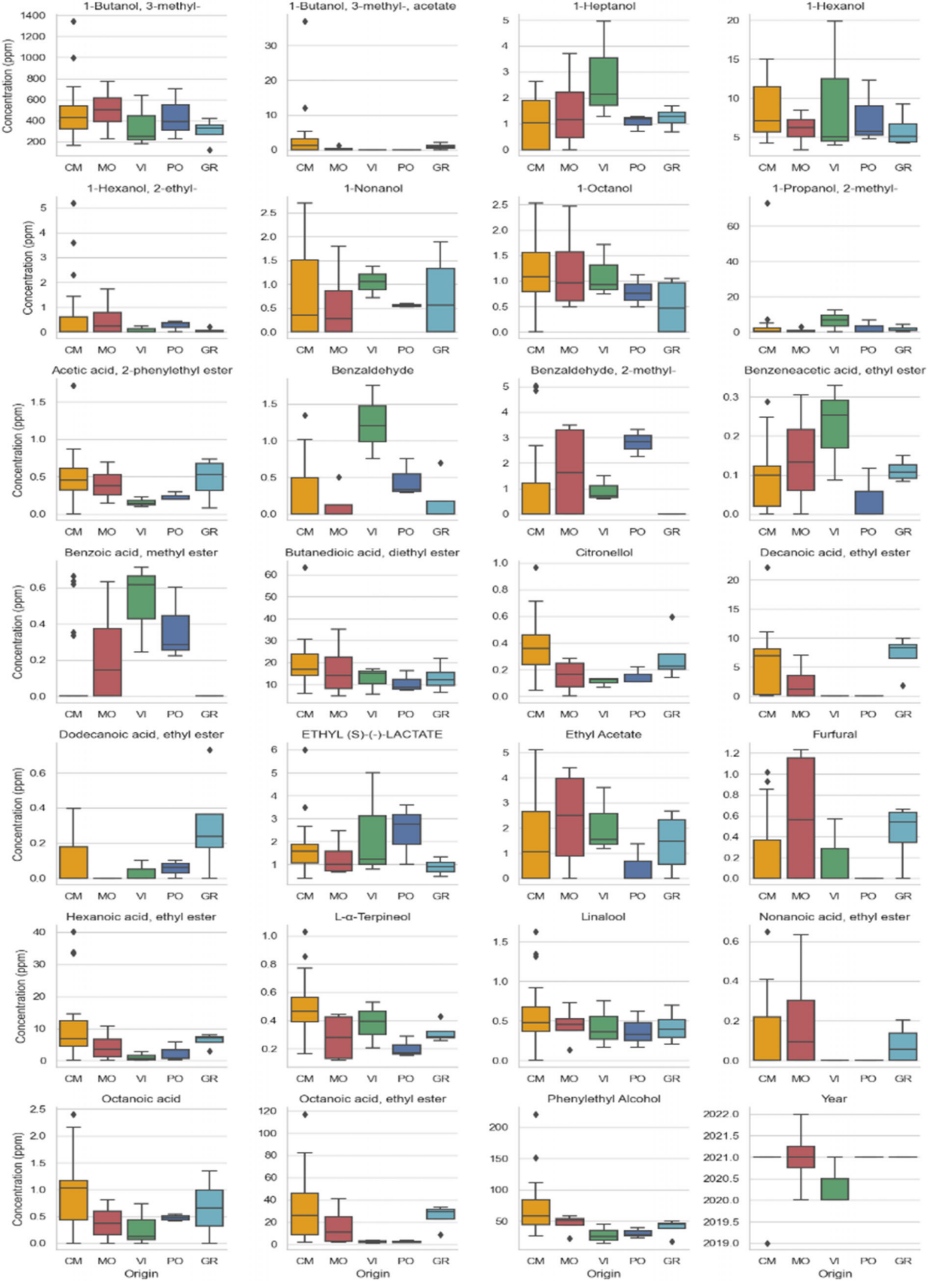
Boxplots of all volatile compounds detected in Ruché wine samples. CM, Castagnole Monferrato; GR, Grana; MO, Montemagno; PO, Portacomaro; VI, Viarigi.

Starting from an initial dataset characterized by 36 samples and 40 variables, a first data filter was applied to make the final data matrix more manageable. Thirteen out of 40 variables were removed because they were not consistently detected in all the samples. The final data matrix included 36 samples and 27 variables (VOCs). A further column indicating the origin (village) was introduced *a posteriori* to make the graphic interpretation based on territory clearer. Figure 1 provides an overview of the concentration (ppm) distribution of all detected analytes based on the wine origin.

**Figure 2:**
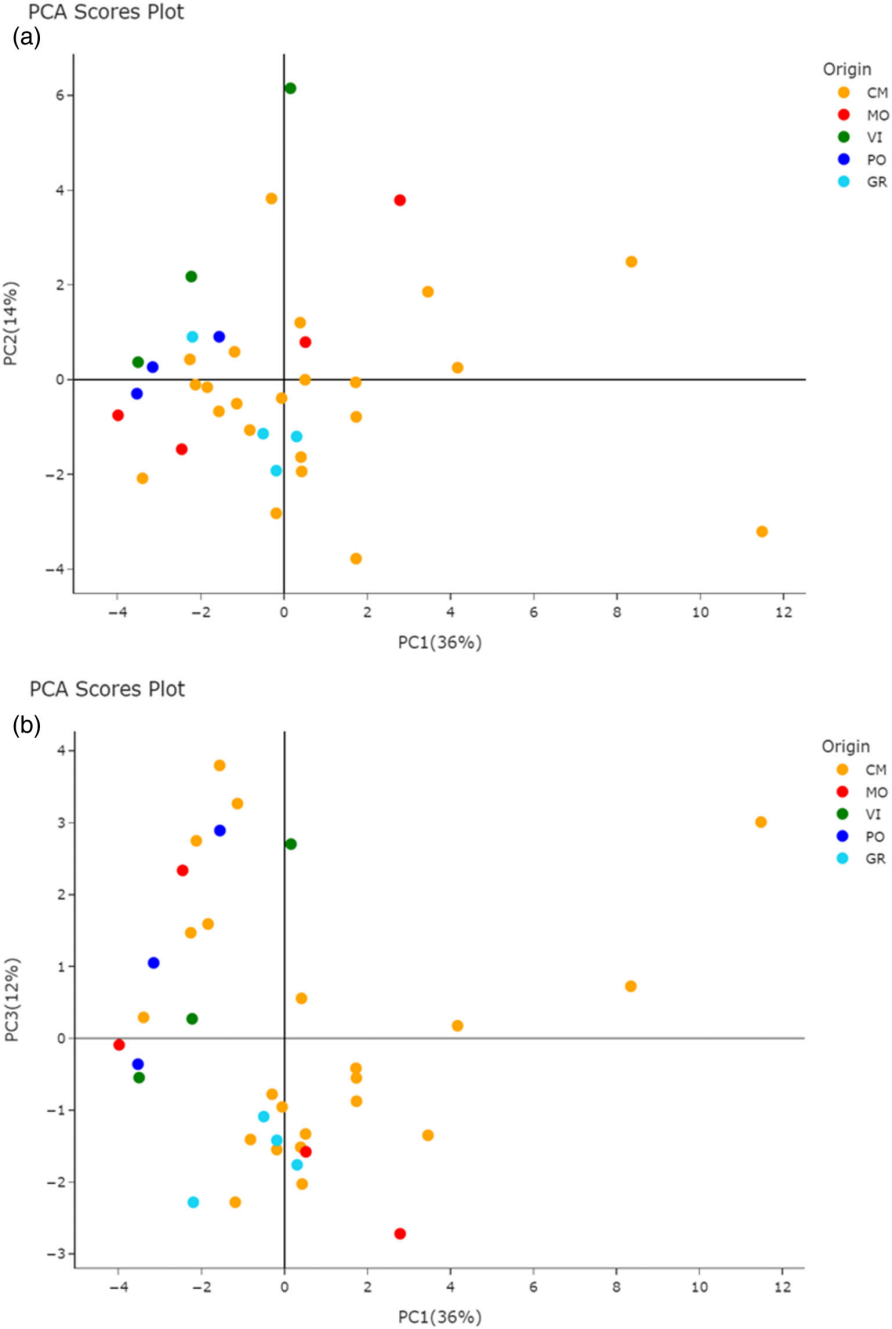
(a) Scores plot showing the projection of the samples on the first two principal components. (b) Scores plot showing the projection of the samples on the first and third principal components. CM, Castagnole Monferrato; GR, Grana; MO, Montemagno; PO, Portacomaro; VI, Viarigi.

Principal component analysis was performed on the final data matrix. The variance explained by the first three principal components is 36%, 14% and 12% respectively, for a total of 62%. The corresponding scores plots are reported in Fig. 2(a) (PC1 vs. PC2) and 2b (PC1 vs. PC3). From Fig. 2(a) it is possible to detect a partial separation between the joined CM and GR samples and the others, in particular VI and PO, along the bisector between the first and third quadrants. The same trend is enhanced in Fig. 2(b): all the samples belonging to VI and PO stand above this bisector whereas those of group GR are located below it, together with the majority of CM samples. Initially, wine samples from CM and GR productions appear similar, likewise VI and PO productions, because their data points are roughly concentrated in the same PC space portion. In contrast, the two sub‐groups show differences attributable to their composition. In the case of MO samples that are equally divided into the two sub‐groups, geographic location appears not to play a role. Instead, the production process adopted by the different wineries and the vintage may be reasonably considered to represent other data influencing factor that should be taken into account for all sample sub‐sets, remembering the restricted geographical area of Ruché wine production (around 80 km^2^). In particular, the possible effect of the vintage was considered. Even though a clear predominance of a specific vintage (2021) was present, no difference was highlighted between 2021 and ‘non‐2021’ wines. The latter were scattered throughout the PCA space (Supporting Information, Fig. S1).

**Figure 3:**
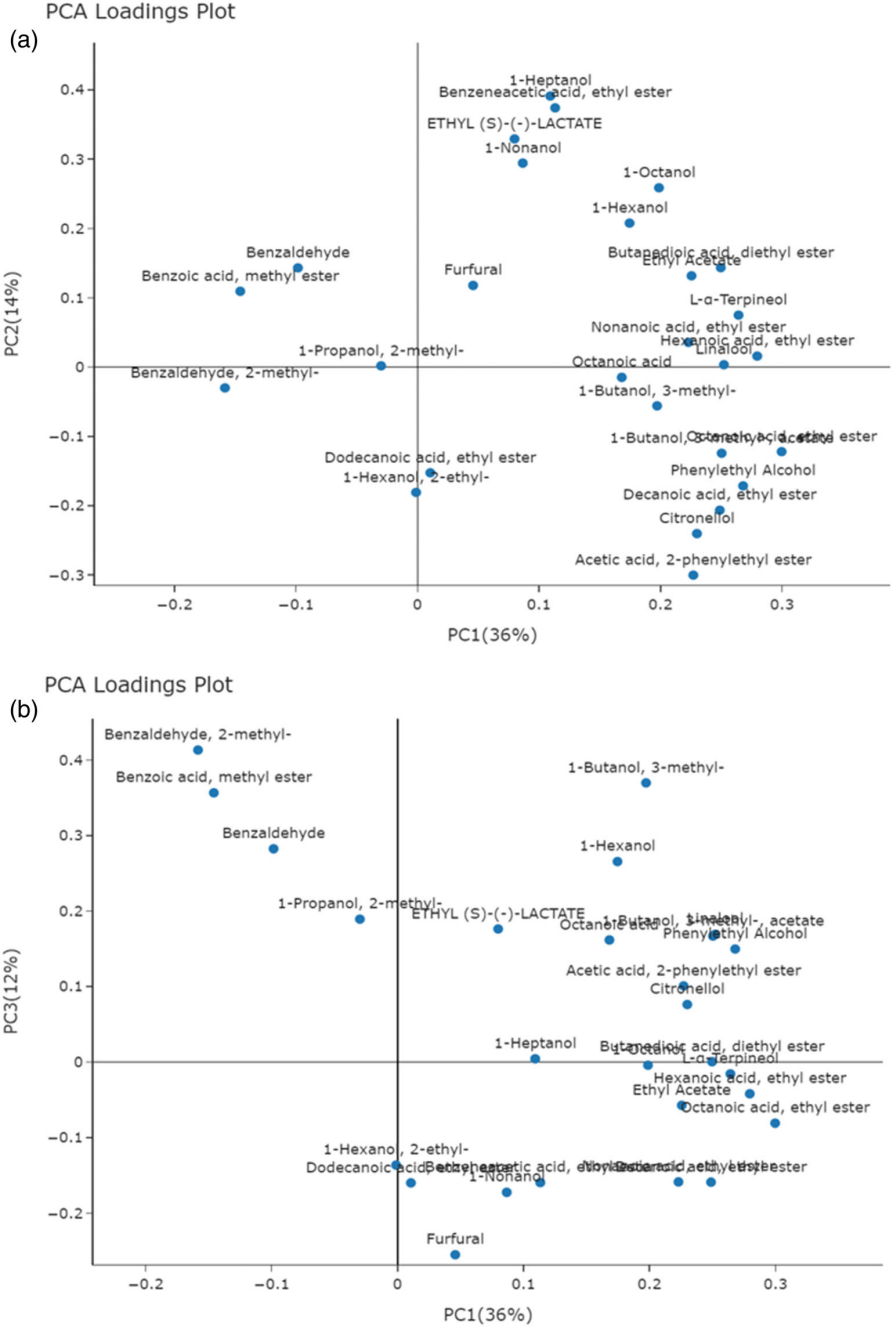
(a) Loadings plot showing the projection of the volatile compounds on the first two principal components. (b) Loadings plot showing the projection of the volatile compounds on the first and the third principal components.

The interpretation of the partial separation shown in Fig. 2 relies on the loadings plots in Fig. 3(a) and (b). In particular, variables with greater importance in describing samples on the score plot (Fig. 2) exhibit their same orientation in the loadings plot (Fig. 3). Consequently, samples above the bisector (e.g., VI and PO) are characterized by high levels of benzaldehyde, 2‐methyl‐benzaldehyde, methyl‐benzoate, and 2‐methyl‐1‐propanol (isobutanol), all located in the second quadrant of Fig. 3. The high abundance of the two aldehydes may be related to the presence of *Botrytis cinerea* spoilage, which is able to transform benzyl alcohol in benzaldehyde.^31^ Notably, both benzaldehyde and methyl benzoate give the wine a marked bitter/green almond hint. Methyl benzoate is not frequently found in wines; in a 2001 paper, Aznar *et al*.^32^ reported the first identification of this component in Spanish red wines from Rioja. Together with benzaldehyde and its methyl derivative, methyl benzoate belongs to the class of benzenoids, reported to provide lesser differentiation among wines produced in the same area and similar procedures.^33^ Asproudi *et al*.^34^ suggested that environmental stress might activate the chemical pathways leading to volatile benzenoids formation in grapes, which also show higher concentrations in grapes from old‐vine vineyards. Accordingly, the websites of some producers located in the PO territory report that their Ruché stems from 40‐year‐old vines, which agrees with the high benzenoids concentration found in them. Isobutanol characterizes VI samples (not PO) with a slightly higher concentration. At high concentration levels, isobutanol and isoamyl alcohol are essentially detrimental to wine quality^35^ An anomalous amount (Fig. 1) of these components was only found in a single CM wine (ru37), suggesting the occurrence of peculiar production conditions (i.e., yeast selection, fermentation conditions, wine blending). The producer's website does not report any information regarding the winemaking procedure, its activity only started in 2020, notably making it the youngest winemaker in the consortium.

The fourth quadrant mostly contains the GR and CM samples, characterized by higher concentrations of several ethyl esters, including octanoic, nonanoic, decanoic, and dodecanoic, together with citronellol and acetic acid, and phenylethyl ester, some of which are particularly low in VI and PO samples. The components depicted in the first quadrant of Fig. 3, basically higher alcohols, such as isoamyl alcohol, 2‐phenylethanol, 1‐hexanol, and 1‐heptanol, do not characterize specific production territories but rather single samples. These compounds bring to the wine flavors, either pleasing or distasteful, which depend strongly on their concentration.

**Figure 4:**
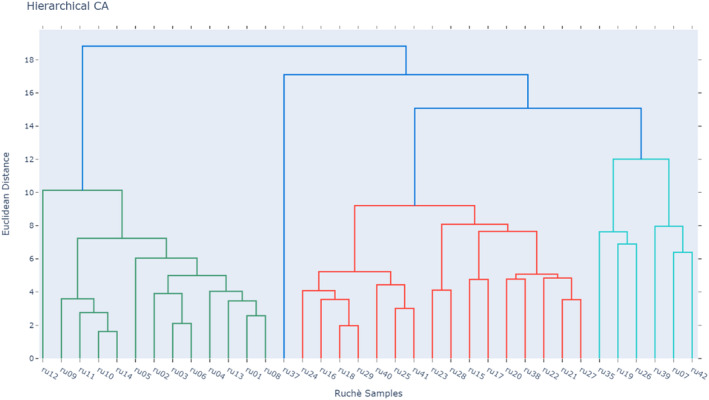
Hierarchical cluster analysis performed on the full dataset, using Ward's method and Euclidean distance.

**Figure 5:**
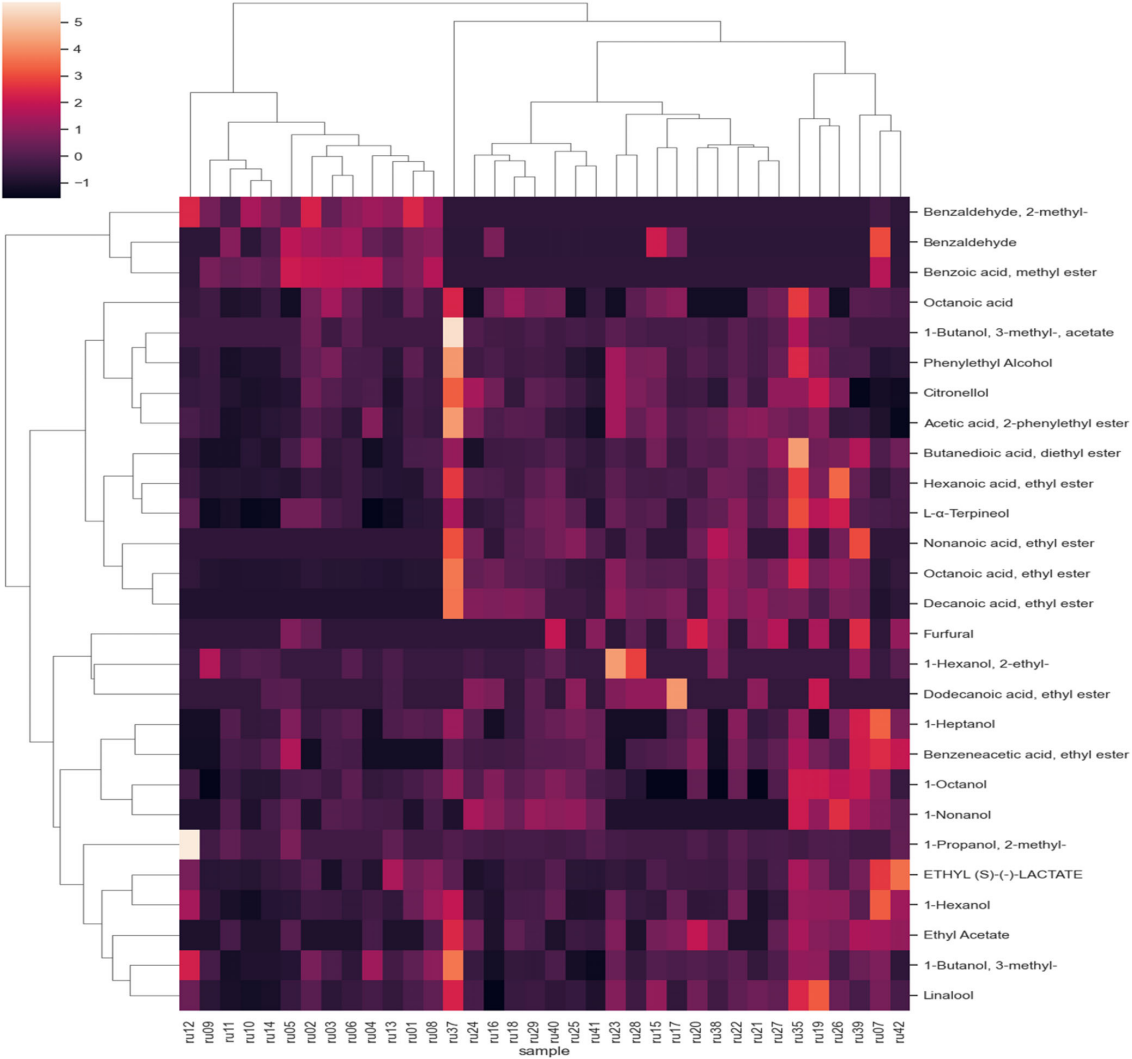
Cluster heat map analysis of volatile organic compounds in Ruché wine samples. The chromatic scale (from low values, dark violet, to high values, skin color) indicates the correlation between the variable concentration and the wine sample.

Further insight into the unsupervised exploration of the data is provided by HCA, from which the dendrogram shown in Fig. 4 is derived. In this plot, the Euclidean distance between couples of samples is reported on the *y*‐axis and the Ward linkage approach is used for their aggregation. The resulting clusters are in full agreement with the sample distribution observed in Fig. 2. It is possible to highlight (i) the presence of the cluster related to the first samples (ru1‐ru14, without ru07), corresponding to the samples above the bisector between first and third quadrant, (ii) an outlier evidenced as a singleton (ru37), (iii) the turquoise cluster that includes all samples characterized by high alcohol and ester concentrations. The heatmap depicted in Fig. 5 completes the information with the VOCs clustering and provides an immediate overview of the volatile composition of all Ruché samples.

**Figure 6:**
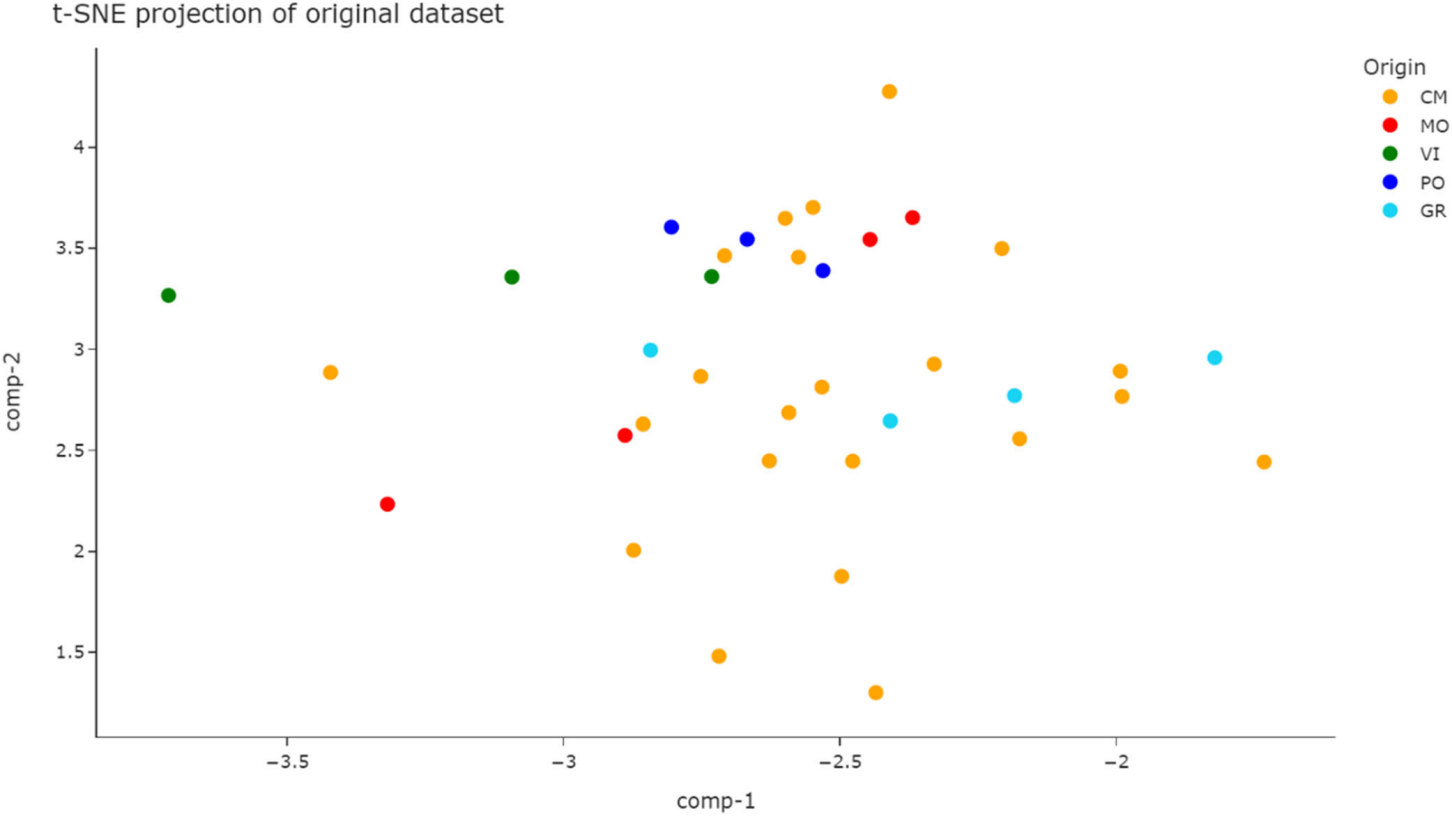
Dimensionality reduction performed by the *t*‐distributed stochastic neighbor embedding approach. CM, Castagnole Monferrato; GR, Grana; MO, Montemagno; PO, Portacomaro; VI, Viarigi.

Finally, Fig. 6 shows the dimensionality reduction performed by t‐SNE. Once again this algorithm underlines the separation already mentioned, endorsing the results provided by the previously unsupervised methods.

## CONCLUSIONS

The aim of this study was to characterize the volatile profile of Ruché, a typical wine from Piedmont, Italy, and to highlight the minute differences in Ruché wine specimens produced in the restricted cultivation area of this native vine variety. This wine features an elevated concentration of higher alcohols and various unusual components, which make Ruché wine popular as a result of the presence of different aromatic combinations. In the present study, the sampling technique allowed the efficient extraction and highlighting of the most abundant semi‐polar aromatic species, namely 2‐phenylethanol (floral nuances), diethyl succinate (fruity), and isoamyl acetate (banana). Conversely, the extraction of polar compounds such as varietal aromas, which are normally less abundant but are peculiar in defining the uniqueness of a wine, was less efficient. Most of the detected aromatic compounds showed an OAV above 1, thus contributing to the complex wine *bouquet*.

Multivariate analysis allowed the identification of small but peculiar differences among the studied samples flavors, despite their homogeneity in terms of vintage and production territory. The present study is likely to provide useful information for producers in order to better characterize their production and improve winemaking procedures.

## FUNDING INFORMATION

Research activities have been conducted within the framework of the Agritech National Research Center and received funding from the European Union Next‐GenerationEU (PIANO NAZIONALE DI RIPRESA E RESILIENZA (PNRR) – MISSIONE 4 COMPONENTE 2, INVESTIMENTO 1.4 – D.D. 1032 17/06/2022, CN00000022).

## CONFLICT OF INTEREST

The authors declare no conflicts of interest.

## AUTHOR CONTRIBUTIONS

**Ciro Orecchio**: conceptualization, methodology, software, investigation, resources, data curation, writing‐original draft preparation, writing‐review and editing. **Roberto Rabezzana**: conceptualization, methodology, investigation, data curation, writing‐original draft preparation, funding acquisition. **Marco Vincenti**: conceptualization, methodology, resources, data curation, writing‐review and editing, funding acquisition. **Elisabetta Bonometti**: formal analysis, investigation. **Enzo Laurenti**: formal analysis, investigation. **Monica Rigoletto**: formal analysis. **Lorenza Operti**: resources, writing‐review and editing.

## Supporting information


**Figure S1.** (a) Scores plot showing the projection of the samples on the first two principal components, in order to evaluate any vintage clustering. (b) Scores plot showing the projection of the samples on the first and third principal components.

## Data Availability

The data supporting the outcomes of this study are available from the corresponding author upon reasonable request.
