# Supplementary material for: Characterization of the aromatic profile of Ruché wine from Piedmont (Italy) with gas chromatography–mass spectrometry and unsupervised machine learning techniques
Source: J Sci Food Agric. 2024 Dec 20;105(6):3193–204. doi: 10.1002/jsfa.14083 (PMC11949860; doi:10.1002/jsfa.14083)
Supplement: Supplementary file 1 — Figure S1. (a) Scores plot showing the projection of the samples on the first two principal components, in order to evaluate any vintage clustering. (b) Scores plot showing the projection of the samples on the first and third principal components. [file JSFA-105-3193-s001.docx]

**SUPPLEMENTARY INFORMATION**

**
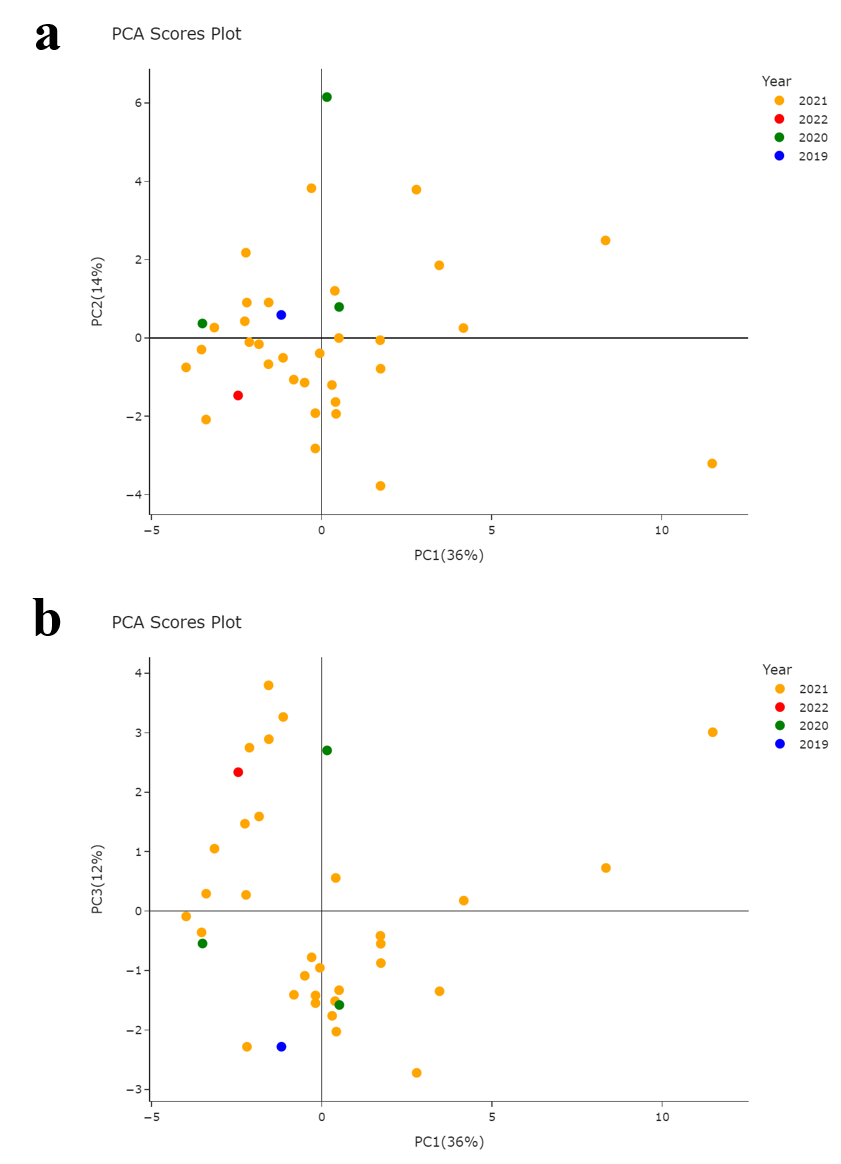
**

**Figure 1S. (a)** Scores plot showing the projection of the samples on the first two principal components, in order to evaluate any vintage clustering. **(b)** Scores plot showing the projection of the samples on the first and third principal components.
